# Adapting Multiple Distributions for Bridging Emotions from Different Speech Corpora

**DOI:** 10.3390/e24091250

**Published:** 2022-09-05

**Authors:** Yuan Zong, Hailun Lian, Hongli Chang, Cheng Lu, Chuangao Tang

**Affiliations:** 1 Key Laboratory of Child Development and Learning Science of Ministry of Education, Southeast University, Nanjing 210096, China; 2 School of Biological Science and Medical Engineering, Southeast University, Nanjing 210096, China; 3 School of Information Science and Engineering, Southeast University, Nanjing 210096, China

**Keywords:** cross-corpus speech emotion recognition, speech emotion recognition, domain adaptation, transfer learning, subspace learning

## Abstract

In this paper, we focus on a challenging, but interesting, task in speech emotion recognition (SER), i.e., cross-corpus SER. Unlike conventional SER, a feature distribution mismatch may exist between the labeled source (training) and target (testing) speech samples in cross-corpus SER because they come from different speech emotion corpora, which degrades the performance of most well-performing SER methods. To address this issue, we propose a novel transfer subspace learning method called multiple distribution-adapted regression (MDAR) to bridge the gap between speech samples from different corpora. Specifically, MDAR aims to learn a projection matrix to build the relationship between the source speech features and emotion labels. A novel regularization term called multiple distribution adaption (MDA), consisting of a marginal and two conditional distribution-adapted operations, is designed to collaboratively enable such a discriminative projection matrix to be applicable to the target speech samples, regardless of speech corpus variance. Consequently, by resorting to the learned projection matrix, we are able to predict the emotion labels of target speech samples when only the source label information is given. To evaluate the proposed MDAR method, extensive cross-corpus SER tasks based on three different speech emotion corpora, i.e., EmoDB, eNTERFACE, and CASIA, were designed. Experimental results showed that the proposed MDAR outperformed most recent state-of-the-art transfer subspace learning methods and even performed better than several well-performing deep transfer learning methods in dealing with cross-corpus SER tasks.

## 1. Introduction

Speech is one of the most natural behaviors through which emotional information is communicated in the daily life of human beings [1,2]. Hence, research into speech emotion recognition (SER), which seeks to enable machines to learn how to automatically understand emotional states, e.g., Happy, Fearful, and Sad, from speech signals, has attracted attention among affective computing, pattern recognition, and speech signal processing research communities. Over recent decades, many well-performing SER methods have been proposed and have achieved promising levels of performance for widely-used speech emotion corpora [3,4,5,6,7,8]. However, the existing SER methods are far from being practically applicable. One of the major reasons is that such methods do not consider real-world scenarios, in which the training and testing speech signals may be recorded by different acoustic sensors. For example, the audio data of EmoDB [9], a widely-used speech emotion corpus, were recorded using a Sennheiser MKH40-P48 microphone and a Tascam DA-P1 portable DAT recorder. However, as for another popular speech emotion corpus, CASIA [10], its samples were recorded using a RODE K2 (a large membrane microphone) and Fireface 800 (sound card). When using these two speech emotion corpora to alternatively serve training and testing purposes, an evident feature distribution mismatch inevitably exists between their corresponding feature sets due to the acoustic sensor difference. Hence, the performance of an initially well-performing SER method will drop significantly.

The above example highlights a challenging, but interesting, task in SER, i.e., cross-corpus SER. Formally, in the task of cross-corpus SER, the training and testing speech sample sets belong to different corpora. The emotion label information of the training sample sets is provided, while the target sample sets’ labels are not entirely given. We need to enable a classifier guided by the source emotion label information to accurately predict the emotions of the unlabeled testing speech samples. Note that, in what follows, we follow the custom in the research concerning transfer learning and domain adaptation [11,12,13], which are closely related to cross-corpus SER, and refer to the training and testing speech samples/signals/corpora/feature sets as the source and target sets, respectively, such that readers can better understand this paper.

In this paper, we try to deal with cross-corpus SER tasks from the perspective of transfer learning and domain adaptation and propose a straightforward transfer subspace learning method called multiple distribution-adapted regression (MDAR). As with most existing transfer subspace learning methods [14,15,16,17,18], MDAR aims to learn a projection matrix to find a common subspace bridging the source and target speech samples from different corpora. However, we pay more attention to designing an emotion wheel knowledge-guided regularization term to help MDAR better eliminate the feature distribution difference between the source and target speech samples. Specifically, instead of directly measuring and improving both corpora’s marginal feature distribution gaps, our MDAR incorporates the idea of joint distribution adaption (JDA) [17] and joint alleviation of marginal distribution mismatch and fine emotion class-aware conditions. More importantly, unlike existing JDA-based methods [16,17,19,20], MDAR extends the JDA operation to a multiple distribution adaption (MDA) method by additionally introducing a well-designed rough emotion class-aware conditional distribution adaption to improve the feature distribution difference alleviation between the speech samples from different corpora. By resorting to MDA, MDAR can learn both corpus invariant and emotion discriminative feature representations for cross-corpus SER.

To evaluate the proposed MDAR, we carried out extensive cross-corpus SER experiments on three widely used speech emotion corpora, including EmoDB [9], eNTERFACE [21], and CASIA [10]. The experimental results showed that, compared with existing state-of-the-art transfer subspace learning, and several well-performing deep transfer learning methods, our MDAR achieved more promising performance when dealing with cross-corpus SER tasks. In summary, the main contributions of this paper are three-fold:

1. We propose a novel transfer subspace learning method called MDAR to deal with cross-corpus SER tasks. The basic idea of MDAR is very straightforward, i.e., learning corpus invariant and emotion discriminative representations for both source and target speech samples belonging to different corpora such that the classifier learning based on the labeled source speech samples is also applicable to predicting the emotions of target speech signals.
2. We present a new distribution difference alleviation regularization term called MDA for MDAR to guide the corpus invariant feature learning for the recognition of the emotions of speech signals. MDA collaboratively aligns marginal, fine emotion class-aware conditional, and rough emotion class-aware feature distributions between source and target speech samples.
3. Three widely used speech emotion corpora, i.e., EmoDB, eNTERFACE, and CASIA, were used to design the cross-corpus SER tasks to evaluate the proposed MDAR. Extensive experiments were conducted to demonstrate the effectiveness and superior performance of MDAR in coping with cross-corpus SER tasks.

The remainder of this paper is organized as follows: Section 2 reviews progress in cross-corpus SER. Section 3 provides details of the proposed MDAR method. In Section 4, extensive cross-corpus SER experiments, conducted to evaluate the proposed MDAR method, are described. Finally, we conclude this paper in Section 5.

## 2. Related Works

In this section, we briefly review recent advances in research concerning cross-corpus SER. To deal with cross-corpus SER tasks, considerable effort has been applied by researchers to focus on solving its key problem, i.e., relieving the feature distribution difference between the source and target speech samples belonging to different corpora. In what follows, we first describe the progress of cross-corpus SER based on transfer subspace learning methods. Moreover, we also introduce recent research into the use of deep transfer learning methods to deal with cross-corpus SER tasks.

### 2.1. Transfer Subspace Learning for Cross-Corpus SER

The earliest investigations into cross-corpus SER may be traced to [22], in which Schuller et al. proposed the adoption of different normalization methods, including speaker normalization (SN), corpus normalization (CN), and speaker-corpus normalization (SCN) to balance the source and target speech corpora. Then, the classifier which absorbs only the emotion discriminant information from the source speech corpus can also be applied to the target speech corpus. Subsequently, transfer subspace learning methods have been used to address the cross-corpus SER problem. For example, Hassan et al. [23] built an importance-weighted support vector machine (IW-SVM) classifier integrating three typical IW methods, i.e., kernel mean matching (KMM) [24], unconstrained least-squares importance fitting (uLSIF) [25], and the Kullback–Leibler importance estimation procedure (KLIEP) [26], to compensate for source speech samples such that the feature distribution gap between two different speech emotion corpora can be better removed. Recently, Song et al. [27] and Zhang et al. [16] designed transfer subspace learning models to learn a shared projection matrix to jointly build the relationship between the emotion labels and transformed speech features, and to align the source and target speech samples’ feature distributions.

### 2.2. Deep Transfer Learning for Cross-Corpus SER

Apart from the above subspace learning methods, inspired by the success of deep transfer learning and deep domain adaptation in many cross-domain visual recognition tasks, researchers have also designed domain invariant deep neural networks to deal with the cross-corpus SER problem. For example, Deng et al. [28,29] proposed a series of unsupervised deep domain adaptation methods using autoencoder (AE) networks instead of projection matrices to seek a common subspace for both source and target speech signals such that their new representations in the common subspace are similarly distributed. Gideon et al. [30] were motivated by the idea of generative adversarial networks (GANs) [31] and presented an adversarial discriminative domain generalization (ADDoG) model to cope with cross-corpus SER tasks. ADDoG consists of three major modules, i.e., a feature encoder, an emotion classifier, and a critic. Among these, the critic is one of the major modules aiming to remove the bias between the source and target speech corpora by estimating their earth-mover or Wasserstein distance. In addition, it is also of note that, unlike most existing methods, ADDoG made use of speech spectrums rather than hand-crafted speech features to serve as the inputs of networks. Hence, it is an end-to-end learning method.

## 3. Proposed Method

### 3.1. Notations

In this section, we address the proposed MDAR method in detail and describe how to use MDAR to deal with cross-corpus SER tasks. To begin with, we give several notations which are needed in formulating MDAR. Suppose we have a set of labeled source speech samples from one corpus whose feature matrix is denoted by $X_{s}\in R^{d\times n_{s}}$, where *d* is the dimension of the speech feature vectors and $n_{s}$ is the source speech sample number. Their corresponding emotion ground truth information is denoted by a label matrix $Y_{s}\in R^{c\times n_{s}}$, where *c* is the emotion class number and its *i*th column $y_{i}={\left[y_{1}^{i},\ldots ,y_{c}^{i}\right]}^{T}$ describes its corresponding speech sample’s emotion information. As for $y_{i}$, only the *j*th entry is set as 1 while the others are set as 0 if this speech sample’s label is the *j*th emotion.

Simliarly, let the target speech feature matrix corresponding to the other corpus and its corresponding unknown label matrix be **$X_{t}\in R^{d\times n_{t}}$** and $Y_{t}\in R^{c\times n_{t}}$, where $n_{t}$ is the target sample number. According to the emotion class, we divide the source and target speech feature matrices $X_{s}$ and $X_{t}$ into $\left\{X_{s_{f}}^{\left(1\right)},\ldots ,X_{s_{f}}^{\left(c\right)}\right\}$ and $\left\{X_{t_{f}}^{\left(1\right)},\ldots ,X_{t_{f}}^{\left(c\right)}\right\}$, where $X_{s_{f}}^{\left(i\right)}$ and $X_{t_{f}}^{\left(i\right)}$ denote the source and target speech feature matrices corresponding to the *i*th emotion among the fine emotion class set {1,…,c}. Accordingly, several fine emotion class feature matrix sets can further merge to obtain the rough emotion class feature matrix set for source and target speech samples, which can be expressed as $\left\{X_{s_{r}}^{\left(1\right)},\ldots ,X_{s_{r}}^{\left(c_{r}\right)}\right\}$ and $\left\{X_{t_{r}}^{\left(1\right)},\ldots ,X_{t_{r}}^{\left(c_{r}\right)}\right\}$, where $X_{s_{r}}^{\left(i\right)}$ and $X_{t_{r}}^{\left(i\right)}$ represent the feature matrices corresponding to the *i*th rough emotion class and $c_{r}$ is the rough emotion class number.

### 3.2. Formulation of MDAR

As described previously, the basic idea of MDAR is to build a subspace learning model to learn emotion discriminative and corpus invariant representations for both source and target speech samples belonging to different corpora. To achieve this goal, we propose to use the label-information-guided feature space to serve as the subspace and then learn a projection matrix to build the relationship between this subspace and the original feature space, which can be formulated as a simple linear regression optimization problem:

(1) $$\begin{array}{c} \min_{U}{∥Y_{s}-U^{T}X_{s}∥}_{F}^{2}, \end{array}$$

where U is such a satisfactory projection matrix and ${∥\cdot ∥}_{F}$ denotes the Frobenius norm of a matrix. Using U, we can easily transform the speech samples from the original feature space to the emotion label space. In other words, this learned projection matrix is endowed with emotion discriminative ability.

Subsequently, we need to further enable the projection matrix U to be robust to the variance of speech corpora such that it is applicable to the problem of cross-corpus SER. To this end, we design a regularization term to help MDAR learn such an expectative projection matrix, whose corresponding optimization problem can be expressed as follows:

(2) $$\begin{array}{cc} \min_{U} & ∥U^{T}{∥}_{2,1}+\lambda_{1}(∥\frac{1}{n_{s}}U^{T}X_{s}1_{s}-\frac{1}{n_{t}}U^{T}X_{t}1_{t}{∥}^{2}\\ & +\sum_{i=1}^{c}∥\frac{1}{n_{s_{f}}^{\left(i\right)}}U^{T}X_{s_{f}}^{\left(i\right)}1_{s_{f}}^{\left(i\right)}-\frac{1}{n_{t_{f}}^{\left(i\right)}}U^{T}X_{t_{f}}^{\left(i\right)}1_{t_{f}}^{\left(i\right)}{∥}^{2}+\sum_{i=1}^{c_{r}}∥\frac{1}{n_{s_{r}}^{\left(i\right)}}U^{T}X_{s_{r}}^{\left(i\right)}1_{s_{r}}^{\left(i\right)}-\frac{1}{n_{t_{r}}^{\left(i\right)}}U^{T}X_{t_{r}}^{\left(i\right)}1_{t_{r}}^{\left(i\right)}{∥}^{2}), \end{array}$$

where $\lambda_{1}$ is a trade-off parameter controlling the balance between different terms, and $1_{s}$, $1_{t}$, $1_{s_{f}}^{\left(i\right)}$, $1_{t_{f}}^{\left(i\right)}$, $1_{s_{r}}^{\left(i\right)}$, and $1_{t_{r}}^{\left(i\right)}$ are all the one-valued vectors, and their dimensions are the numbers of source and target samples denoted by $n_{s}$ and $n_{t}$, target and target samples corresponding to *i*th fine emotion class denoted by $n_{s_{f}}^{\left(i\right)}$ and $n_{t_{f}}^{\left(i\right)}$, and source and target samples corresponding to *i*th rough emotion class denoted by $n_{s_{r}}^{\left(i\right)}$ and $n_{t_{r}}^{\left(i\right)}$, respectively.

From Equation (2), it is clear that the objective function designed for the corpus robustness of the projection matrix consists of a $l_{2,1}$ norm and a combination of marginal, fine emotion class-aware conditional, and rough emotion class-aware conditional distributions aligned functions with respect to U, respectively. These two terms correspond to two aspects of our efforts regarding MDAR:

1. $∥U^{T}{∥}_{2,1}$ can be called the feature selection term. Minimizing $∥U^{T}{∥}_{2,1}$ helps the MDAR learn a row-sparse projection matrix, which suppresses the speech features contributing less to the distinction of different emotions, while highlighting the features contributing most to distinction.
2. The other aspect is the multiple distribution adaption (MDA), which corresponds to the resting three terms. Among these, the first two terms are so-called joint distribution adaptions (JDA) [16,17,19,20]. JDA is a combination of the marginal distribution adaption and the fine emotion class-aware conditional adaption and has been demonstrated the effectiveness in coping with domain adaptation and other cross-domain recognition tasks. Our MDA can be viewed as an extension of JDA incorporating an additional rough emotion class-aware conditional distribution-adapted term, which enables further enhancement of the corpus invariant ability of the proposed MDAR.

Finally, by combining Equations (1) and (2), we arrive at the eventual optimization problem of the proposed MDAR method, which can be formulated as follows:

(3) $$\begin{array}{c} \min_{U}∥Y_{s}-U^{T}X_{s}{∥}_{F}^{2}+\lambda ∥U^{T}{∥}_{2,1}+\mu (∥\frac{1}{n_{s}}U^{T}X_{s}1_{s}-\frac{1}{n_{t}}U^{T}X_{t}1_{t}{∥}^{2}\\ +\sum_{i=1}^{c}∥\frac{1}{n_{s_{r}}^{\left(i\right)}}U^{T}X_{s_{f}}^{\left(i\right)}1_{s_{f}}^{\left(i\right)}-\frac{1}{n_{t_{f}}^{\left(i\right)}}U^{T}X_{t}^{\left(i\right)}1_{t_{f}}^{\left(i\right)}{∥}^{2}+\sum_{i=1}^{c_{r}}∥\frac{1}{n_{s_{r}}^{\left(i\right)}}U^{T}X_{s_{r}}^{\left(i\right)}1_{s_{r}}^{\left(i\right)}-\frac{1}{n_{t_{r}}^{\left(i\right)}}U^{T}X_{t_{r}}^{\left(i\right)}1_{t_{r}}^{\left(i\right)}{∥}^{2}), \end{array}$$

where λ and $\mu =\lambda \times \lambda_{1}$ are the trade-off parameters to balance all the terms.

### 3.3. Disturbance Strategy for Constructing Rough Emotion Groups in MDA

**Figure 1:**
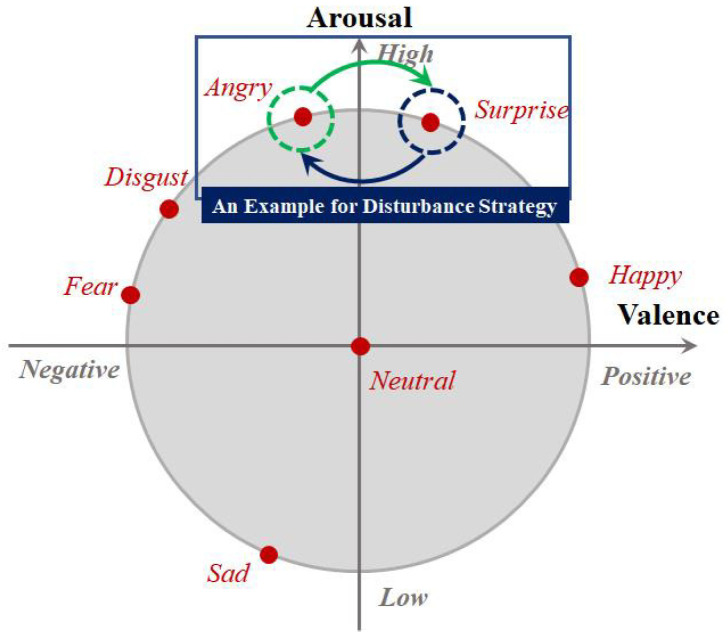
The 2D arousal-valence emotion wheel proposed by Yang et al. [32]. This is a reduced version involving only the emotions used in this paper.

The major inspiration for designing the rough emotion class-aware conditional distribution adapted term to obtain MDA was the recent work of [32], in which a modified 2D arousal-valence emotion wheel consisting of two dimensions, i.e., valence and arousal, is presented. To better understand our motivation, we repost Yang et al.’s emotion wheel in Figure 1. From Figure 1, it is clear that each typical discrete emotion, e.g., Angry, Happy, and Surprise, can be mapped to one point in the emotion wheel based on its corresponding valence and arousal degrees. As the emotion wheel shows, there is an intrinsic distance between two emotions according to their positions on the emotion wheel. Several typical emotions, e.g., Fear vs. Disgust, and Surprise vs. Happy, are very similar and difficult to distinguish from their distance measured with respect to the valence and arousal. In other words, it may be hard to directly align the fine class-aware conditional distribution associated with these emotions due to the unavailability of target speech sample emotion labels. Although we can predict their pseudo emotion labels to calculate statistics for the fine class-aware conditional distribution, the emotion discriminative ability of MDAR is limited in the initial iterations of optimization.

To relieve this tension, in this paper, we introduce the rough emotion class-aware conditional distribution-adapted term and present a disturbance strategy to construct its rough emotion class groups. Specifically, along the valence dimension, we first divide the emotions into two rough emotion class groups including Positive-Valence (Surprise, Happy, and Neutral) and Negative-Valence (Angry, Disgust, Fear, and Sad). Then, regarding the specific cross-corpus SER task, we make several modifications to the original rough emotion groups to break the inseparability of some emotions which have a close distance with respect to the degree of valence and arousal. For example, we can switch Angry and Surprise for High-Valence and Low-Valence groups. Finally, following the modified mixed emotion groups, we calculate the rough emotion class-aware conditional distribution-adapted term $\sum_{i=1}^{c_{r}}{∥\frac{1}{n_{s}}U^{T}X_{s_{r}}^{\left(i\right)}1_{s_{r}}^{\left(i\right)}-\frac{1}{n_{t}}U^{T}X_{t_{r}}^{\left(i\right)}1_{t_{r}}^{\left(i\right)}∥}^{2}$.

Note that, introducing the above rough emotion class-aware conditional distribution-adapted term under the disturbance strategy for MDAR has two expectative benefits. First, the modification of the mixed emotion groups alleviates the inseparability of the emotion elements in Positive-Valence or Negative-Valence groups and, hence, assists fine emotion class-aware conditional distribution adaption in MDAR. Second, unlike the fine emotion class-aware conditional distribution adaption, performing a rough adaption does not require over-precise target pseudo-labels, which affects the fine emotion class-aware conditional distribution adaption. However, the proposed rough adaption does not have this drawback because it only needs rough emotion labels of target speech samples, the prediction of which is an easier task.

### 3.4. Predicting the Target Emotion Label Using MDAR

Once the optimal projection matrix of MDAR denoted by $\hat{U}$ is learned, we are able to predict the emotion label of the target speech samples according to the following criterion:

(4) $$\begin{array}{c} \mathrm{emo}\_\mathrm{label}=\arg\max_{i}y_{t}^{te}\left(i\right). \end{array}$$

Note that $y_{t}^{te}$ denotes the target emotion label vector and can be computed by $y_{t}^{te}={\hat{U}}^{T}x_{t}^{te}$, where $x_{t}^{te}$ is its corresponding feature vector and $y_{t}^{te}\left(i\right)$ is its *i*th entry.

### 3.5. Optimization of MDAR

The optimization of MDAR can be solved by the alternated direction method (ADM) and inexact augmented Lagrangian multiplier (IALM) [33]. Specifically, we first initialize the projection matrix U and then repeat the following two major steps until convergence:

1. Predict the target emotion labels based on the projection matrix U and Equation (4). Then compute the original marginal and two aware conditional **feature** distribution gaps denoted by $\Delta_{m}$, $\Delta_{f}^{\left(i\right)}$, and $\Delta_{r}^{\left(i\right)}$ according to the predicted target emotion labels using the following Equations (5)–(7):
  (5) $$\begin{array}{c} \Delta_{m}=\frac{1}{n_{s}}X_{s}1_{s}-\frac{1}{n_{t}}X_{t}1_{t}. \end{array}$$
  (6) $$\begin{array}{c} \Delta_{f}^{\left(i\right)}=\frac{1}{n_{s_{f}}^{\left(i\right)}}X_{s_{f}}^{\left(i\right)}1_{t_{f}}^{\left(i\right)}-\frac{1}{n_{t_{f}}^{\left(i\right)}}X_{t_{f}}^{\left(i\right)}1_{t_{f}}^{\left(i\right)}, \end{array}$$
  where i={1,…,c}.
  (7) $$\begin{array}{c} \Delta_{r}^{\left(i\right)}=\frac{1}{n_{s_{r}}^{\left(i\right)}}X_{s_{r}}^{\left(i\right)}1_{s_{r}}^{\left(i\right)}-\frac{1}{n_{t_{r}}^{\left(i\right)}}X_{t_{r}}^{\left(i\right)}1_{t_{r}}^{\left(i\right)}, \end{array}$$
  where $i=\{1,\ldots ,c_{r}\}$.
2. Solve the following optimization problem:
  (8) $$\begin{array}{c} \min_{U}∥\left[Y_{s},0\right]-U^{T}\left[X_{s},\sqrt{\mu }\Delta \right]{∥}_{F}^{2}+\lambda {∥U^{T}∥}_{2,1}, \end{array}$$
  where $0\in R^{c\times (c+c_{r}+1)}$ is a zero matrix, and $\Delta =\left[\Delta_{m},\Delta_{f}^{\left(1\right)},\ldots ,\Delta_{f}^{\left(c\right)},\Delta_{r}^{\left(1\right)},\ldots ,\Delta_{f}^{\left(c_{r}\right)}\right]\in R^{d\times (c+c_{r}+1)}$.

As for Equation (8), IALM can be used to efficiently optimize it. More specifically, we introduce an auxiliary variable P satisfying P=U. Thus, we can convert the original optimization problem to a constrained problem as follows:

(9) $$\begin{array}{c} \min_{U,P}∥L-P^{T}{Z∥}_{F}^{2}+\lambda {∥U^{T}∥}_{2,1},\;s.t.\;P=U, \end{array}$$

where $L=[Y_{s},0]$ and $Z=[X_{s},\sqrt{\mu }\Delta ]$.

Subsequently, we can write its corresponding Lagrangian function as follows:

(10) $$\begin{array}{c} L\left(U,P,T,\kappa \right)=∥L-P^{T}{Z∥}_{F}^{2}+\lambda {∥U^{T}∥}_{2,1}\;\;\;\;\;\;\;\;\;\;\;\;\;\;\;\;\\ +Tr\left[T^{T}\left(P-U\right)\right]+\frac{\kappa }{2}{∥P-U∥}_{F}^{2}, \end{array}$$

where **Tr(·) denotes the trace of a square matrix**, T is the multiplier matrix and κ is the trade-off parameter. By alternatively minimizing the Lagrangian function with respect to the variables, we can obtain the optimal U. We summarize the detailed updating rules in Algorithm 1.
**Algorithm 1** Complete updating rule for learning the optimal U in Equation (10).**Repeat the following steps until convergence:**Fix U, T, and κ, update P: $\min_{P}∥L-P^{T}{Z∥}_{F}^{2}+Tr\left(T^{T}P\right)+\frac{\kappa }{2}{∥P-U∥}_{F}^{2}$, which results in $P={\left(2ZZ^{T}+\kappa I\right)}^{-1}\left(\kappa U-T-ZL^{T}\right)$.Fix P, T, and κ, update U: $\min_{U}\frac{\lambda }{\kappa }∥U^{T}{∥}_{2,1}+\frac{1}{2}{∥U^{T}-\left(P^{T}+\frac{T^{T}}{\kappa }\right)∥}_{F}^{2}$, whose solution is obtained by$c_{i}=\frac{∥p_{i}+\frac{t_{i}}{\kappa }∥-\frac{\lambda }{\kappa }}{∥p_{i}+\frac{t_{i}}{\kappa }∥}\left(p_{i}+\frac{t_{i}}{\kappa }\right)$, if $\frac{\lambda }{\kappa }<∥p_{i}+\frac{t_{i}}{\kappa }∥$, where $p_{i}$ and $t_{i}$ are the *i*th row of P and T, respectively. Otherwise, $c_{i}=0$.Update T and κ: T=T+κ(P−U), and $\kappa =\min\{\rho \kappa ,\kappa_{max}\}$.Check convergence: ${∥P-U∥}_{F}<\epsilon$.

## 4. Experiments

### 4.1. Speech Emotion Corpora and Experimental Protocol

In this section, we describe cross-corpus SER experiments to evaluate the proposed MDAR method. In what follows, we give the detail of the evaluation experiments.

1. **Speech Emotion Corpora**: Three widely-used speech emotion corpora, i.e., EmoDB (Berlin) [9], eNTERFACE [21], and CASIA [10], were adopted to design cross-corpus SER tasks. EmoDB is a German corpus and was collected by Burkhardt et al. from TU Berlin, Germany. It consists of 535 acted speech samples from 10 speakers, including five females and five males. Each speech sample is assigned one of seven basic emotion labels, i.e., Neutral(NE), Angry(AN), Fear(FE), Happy(HA), Sad(SA), Disgust(DI), and Boredom. eNTERFACE is an English audio-visual emotion database consisting of 42 speakers from 14 different nationalities. The emotions involved are AN, DI, FE, HA, SA, and Surprise(SU). In the experiments, we only adopted its audio subset. CASIA is a Chinese speech emotion corpus designed by the Institute of Automation, Chinese Academy of Science, China. It includes 1200 speech samples covering six basic emotions, i.e., AN, SA, FE, HA, NE, and SU.
2. **Task Detail**: We used two of the above speech emotion corpora to serve as the source and target corpora, alternatively, and thus derived six typical cross-corpus SER tasks, i.e., B→E, E→B, B→C, C→B, E→C, and C→E, where *B*, *E*, and *C* are short for EmoDB, eNTERFACE, and CASIA, and the left and right corpora of the arrow correspond to the source and target corpora, respectively. It is of note that, since these corpora have different emotions, in each cross-corpus SER task, we extracted speech samples sharing the same emotion labels to ensure label consistency. The detailed sample statistical information of the selected speech emotion corpora is given in Table 1.
3. **Performance Metric**: As for the performance metric, the unweighted average recall (UAR) [22], defined as the accuracy per class averaged by the total emotion class number, was chosen.

### 4.2. Comparison Methods and Implementation Detail

For comparison, we included recent well-performing transfer subspace learning methods, i.e., transfer component analysis (TCA) [14], geodesic flow kernel (GFK) [15], subspace alignment (SA) [34], domain-adaptive subspace learning (DoSL) [35], and joint distribution adaptive regression (JDAR) [16]. Linear SVM was used as the classifier and we report its results for all the cross-corpus SER tasks to serve as the baseline. Since subspace learning methods are not end-to-end methods, they need a hand-crafted speech feature set to describe speech signals. In the experiments, we adopted IS09 [36] and IS10 [37] feature sets provided by the INTERSPEECH 2009 Emotion Challenge and the INTERSPEECH 2010 Paralinguistic Challenge, respectively, for all the subspace learning methods. The IS09 feature set consists of 384 elements produced by 32 low-level descriptors (LLDs), e.g., fundamental frequency (F0), Mel-frequency cepstrum coefficient (MFCC), their first-order difference, and their 12 corresponding functions, e.g., mean, maximal, and minimal value. Compared with IS09, the IS10 feature set contains more LLDs and functions such that its element number increases to 1582. Both feature sets can be conveniently extracted using the openSMILE toolkit [38]; detailed information is available in [36,37].

Furthermore, we also compared our MDAR method with several recent state-of-the-art deep transfer learning methods including the deep adaptation network (DAN) [39], the domain-adversarial neutral network (DANN) [40], deep-CORAL [41], the deep subdomain adaptation network (DSAN) [42], and the deep transductive transfer regression network (DTTRN) [20]. For these deep learning methods, AlexNet was chosen as the CNN backbone and we also used AlexNet to conduct the experiments to serve as the baseline. The speech spectrums served as the network inputs instead of the hand-crafted speech feature sets. Specifically, the frame size and overlap were first set as 350 and 175 sampling points, respectively. Then, for each speech signal, all the frames were windowed using the Hamming function and subsequently transformed to individual spectrums by resorting to Fourier transformation. Finally, these individual spectrums composed the spectrum of the speech signal. Note that due to the unavailability of target label information in cross-corpus SER, a cross-validation method cannot be used to determine the optimal hyper-parameters for all the methods. Hence, following most existing studies [16,35,39,42], in our experiments, we searched the hyper-parameters for all the methods from a preset interval and then reported their best UAR corresponding to the best optimal hyper-parameter. The details of the hyper-parameter setting for all the transfer learning methods were as follows:

1. **TCA**, **GFK**, and **SA**: For these three methods, the hyper-parameter, i.e., the reduced dimension, needed to be set. In the experiments, we searched it from $\left[5:5:d_{max}\right]$, where $d_{max}$ is the maximal dimension.
2. **DoSL** and **JDAR**: DoSL and JDAR have two trade-off parameters controlling the balance between the original loss function and two regularization terms, in which one corresponds to the sparsity and the other corresponds to feature distribution adaption. We searched them both from [5:5:200] in the experiments.
3. **DAN** and **DSAN**: DAN and DSAN both have a trade-off parameter to balance the original loss and the MMD regularization term. In the experiments, we set it by searching from {0.001,0.005,0.01,0.05,0.1,0.5}.
4. **DANN**: As for DANN, it also has only one trade-off parameter. We searched it from the parameter set {0.001,0.003,0.005,0.01,0.05,0.1,0.5} throughout the experiments.
5. **DTTRN**: Since the protocol in [20] was identical to ours, we used the results reported in their experiments for comparison.
6. **MDAR**: Similar to DoSL and JDAR, our MDAR also had two hyper-parameters, i.e., λ and μ. They were used to control the balance between the original regression loss function and the two regularization terms, including the feature selection and feature distribution difference alleviation terms. In the experiments, they were also both searched from the parameter interval [5:5:200]. In addition, the rough emotion class number $c_{r}$ was set to 2 (High-Valence and Low-Valence). The disturbance strategy for the two mixed rough emotion groups was performed as follows: Reassign Disgust from the Low-Valence group to the High-Valence group for B→E and E→B, and Fear from the Low-Valence group to the High-Valence group for B→C and C→B. Switch Angry and Surprise for E→C and C→E.

### 4.3. Results and Discussion

#### 4.3.1. Comparison with Transfer Subspace Learning Methods

The experimental results are shown in Table 2, Table 3 and Table 4. Among these, Table 2 and Table 3 correspond to the comparison among the transfer subspace learning methods using IS09 and IS10 as the feature sets, respectively. From Table 2 and Table 3, several interesting observations can be made:

First, it is clear that the proposed MDAR method achieved the best UAR averaged by the results of all the six cross-corpus SER tasks among all the transfer subspace learning methods when using both IS09 and IS10 feature sets to describe the speech signals. Specifically, the average UAR achieved by our MDAR reached 42.26% and 36.69% in the experiments using IS09 and IS10 as the feature sets, respectively, with promising increases of 0.50% and 0.42% over the second best results (41.76% obtained by JDAR [16] + IS10 and 36.33% obtained by SA [34] + IS09). This indicates that our MDAR demonstrated superior overall performance compared to recent state-of-the-art transfer subspace learning methods when dealing with cross-corpus SER tasks.

Second, it was also evident that, using IS10 as the speech feature set, our MDAR achieved more promising results in terms of UAR than all the comparison methods for the four cross-corpus SER tasks (B→E, E→B, B→C, and E→C) among all the six tasks. Although in the resting tasks our MDAR did not beat the other transfer subspace learning methods, the performance of MDAR was very competitive against the best-performing transfer subspace learning methods, e.g., 37.30% (MDAR) vs. 37.58% (JDAR) in the task of B→E.

Last, but not least, from the comparison between Table 2 and Table 3, it is clear that the performance of all the transfer subspace learning methods varied with respect to the feature set used to describe speech signals. Specifically, the IS10 feature set included more low-level acoustic descriptors and statistical functions than IS09, which provided more emotion discriminative information when recognizing the emotions of speech signals. Hence, the performance of all the transfer subspace learning methods with the IS10 feature set increased remarkably compared to IS09. This remarkable performance increase indicates that, when dealing with cross-corpus SER tasks, the capacity of the hand-crafted speech feature set chosen to describe the speech signals is very important for the transfer of subspace learning methods.

#### 4.3.2. Comparison with Deep Transfer Learning Methods

Table 4 shows the comparison between our MDAR and several recent state-ot-the-art deep transfer learning methods. From Table 4, it can be seen that, in terms of the average UAR, all the deep transfer learning methods outperformed our MDAR using IS09 as the feature set to describe the speech signals. However, when using the IS10 feature set, the performance of our MDAR increased from 36.69% to 42.26% in terms of the average UAR, beating the deep transfer learning methods. More importantly, our MDAR, together with the IS10 feature set, showed superior performance compared with the comparison deep learning methods in five of six cross-corpus SER tasks. These observations further confirmed the effectiveness and satisfactory performance of the proposed MDAR in coping with cross-corpus SER tasks, which would otherwise lose to the deep transfer learning methods if the hand-crafted speech feature set has adequate ability to describe the speech signals adopted.

#### 4.3.3. Going Deeper into Disturbance Strategy in MDAR

As Equation (2) shows, our MDAR absorbs the knowledge of the emotion wheel to design a rough emotion class-aware conditional distribution-adapted term to help corpus-invariant feature learning. In this distribution-adapted term, two rough emotion groups are obtained in advance, according to the valence dimension under the guidance of the disturbance strategy, i.e., switching several emotion elements in both groups. Therefore, it is interesting to consider whether the proposed strategy (denoted by the *Proposed Modification*) is effective for improving MDAR in coping with cross-corpus SER tasks. To this end, we conducted additional experiments choosing tasks using the IS09 feature set as the representatives, and then adopted the original valence-based rough emotion groups to compute this well-designed term (denoted by the *Original Version*) for MDAR. Table 5 presents the experimental results. From Table 5, it can be seen that MDAR achieved better performance when using the proposed disturbance strategy to modify the rough emotion groups and to compute its corresponding conditional distribution-adapted term compared with using the original method.

#### 4.3.4. Sensitivity Analysis of Trade-Off Parameters in MDAR

**Figure 2:**
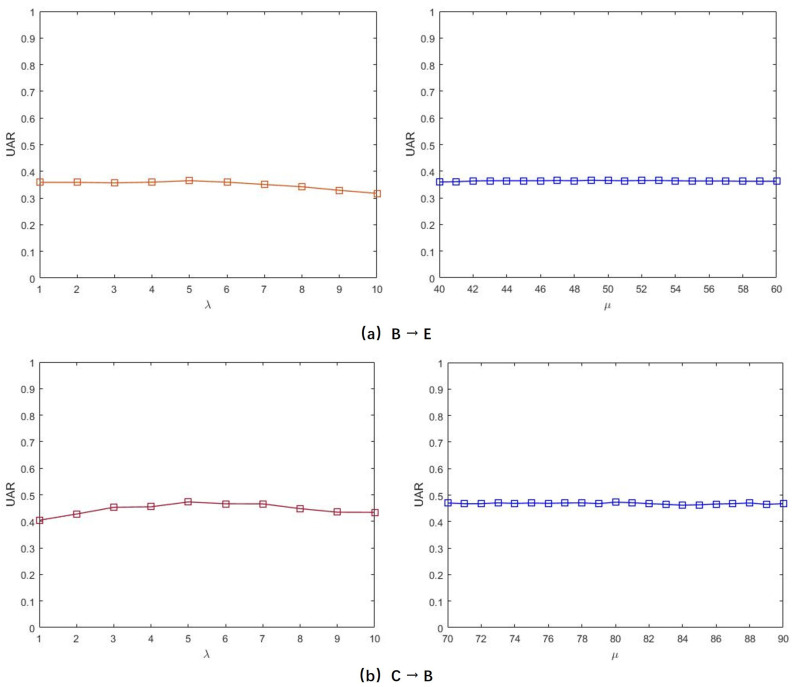
Experimental results of trade-off parameter sensitivity analysis for MDAR, where the λ and μ are set as λ=[1:10], μ=50 for the left and λ=5, μ=[1:10] for the right in (**a**), and λ=[1:10], μ=80 for the left and λ=5, μ=[70:90] for the right in (**b**).

From the optimization problem of MDAR shown in Equation (3), it is known that our MDAR has two major trade-off parameters, including λ and μ, controlling the balance between the original regression loss and the distribution-adapted regularization terms. This generates an interesting problem, i.e., how the performance of the proposed MDAR changes with respect to these two parameters. To investigate this, we conducted additional experiments choosing the tasks B→E and C→B, using the IS09 feature set as the representatives. Specifically, we alternatively fixed one parameter at the optimal value and varied the other for a parameter interval centered at its optimal value, and then performed MDAR for each task. The experimental results are shown in Figure 2, in which the fixed parameter and the varying parameter interval are also provided. From Figure 2, it is clear that the performance of the proposed MDAR varied slightly with respect to the change in both trade-off parameters, which indicates that our MDAR was less sensitive to the choice of its trade-off parameters.

## 5. Conclusions

In this paper, we investigated the problem of cross-corpus SER and proposed a novel effective transfer subspace learning method called MDAR. Unlike most existing transfer subspace learning methods, the proposed MDAR absorbs the emotion wheel knowledge and adopts a well-designed distribution-adapted regularization term which considers the marginal distribution adaption and two-scale emotion-aware conditional adaption to jointly alleviate the feature distribution mismatch between the source and target speech corpora. Extensive cross-corpus SER experiments were carried out to evaluate the performance of the proposed MDAR method. The experimental results demonstrated the effectiveness of MDAR and its superior performance over recent state-of-the-art transfer subspace learning methods, including several high-performing deep transfer learning methods, in coping with cross-corpus SER tasks.

## Figures and Tables

**Table 1:** The sample statistics of the selected speech corpora used in cross-corpus SER tasks.

Tasks	Speech Corpus (# Samples from Each Emotion)	#Sample
B→E	EmoDB (AN: 127, SA: 62, FE: 69, HA: 71, DI: 46)	375
E→B	eNTERFACE (AN: 211, SA: 211, FE: 211, HA: 208, DI: 211)	1052
B→C	EmoDB (AN: 127, SA: 62, FE: 69, HA: 71, NE: 79)	408
C→B	CASIA (AN: 200, Sad: 200, FE: 200, HA: 200, NE: 200)	1000
E→C	eNTERFACE (AN: 211, SA: 211, FE: 211, HA: 208, SU: 211)	1052
C→E	CASIA (AN: 200, SA: 200, FE: 200, HA: 200, SU: 200)	1000

**Table 2:** The comparison results among all the transfer subspace learning methods of using IS09 as feature set, in which the best results are highlighted in bold.

Method	B→ E	E→B	B→C	C→B	E→C	C→E	Average
SVM	28.93	23.58	29.60	35.01	26.10	25.14	28.06
TCA	30.52	**44.03**	33.40	45.07	31.10	**32.32**	36.07
GFK	32.11	42.48	33.10	48.08	**32.80**	28.13	36.17
SA	33.50	43.89	**35.80**	**49.03**	32.60	28.17	36.33
DoSL	36.12	38.95	34.40	45.75	30.40	31.59	36.20
JDAR	36.33	39.97	31.10	46.29	32.40	31.50	36.27
**MDAR**	**36.52**	40.29	33.10	47.32	31.70	31.21	**36.69**

**Table 3:** The comparison results among all the transfer subspace learning methods of using IS10 as feature set, in which the best results are highlighted in bold.

Method	B→ E	E→B	B→C	C→B	E→C	C→E	Average
SVM	34.50	28.13	35.30	35.29	24.30	26.81	30.73
TCA	32.60	44.53	40.50	**51.47**	33.20	29.77	38.68
GFK	36.01	40.11	40.00	45.93	33.00	29.09	37.35
SA	35.65	43.92	37.50	47.06	32.10	30.61	37.80
DoSL	36.82	43.33	36.80	48.45	35.60	33.91	39.15
JDAR	37.95	47.80	42.70	48.97	35.60	**37.58**	41.76
**MDAR**	**38.90**	**48.95**	**43.00**	49.52	**35.80**	37.30	**42.26**

**Table 4:** The comparison results between our MDAR of using IS09 and IS10 as feature sets and all the deep transfer learning methods, in which the best results are highlighted in bold.

Method	B→ E	E→B	B→C	C→B	E→C	C→E	Average
AlexNet	29.49	31.03	33.20	41.91	27.80	27.25	31.78
DAN	36.13	40.41	39.00	49.85	29.00	31.47	37.64
DANN	33.38	43.68	39.20	53.71	29.80	29.25	38.05
Deep-CORAL	35.03	43.38	38.30	48.28	31.00	30.89	37.81
DSAN	36.25	46.90	40.30	50.69	28.70	32.61	39.41
DTTRN	37.70	48.20	40.40	**55.20**	31.20	33.60	41.10
**MDAR + IS09**	36.52	40.29	33.10	47.32	31.70	31.68	36.69
**MDAR + IS10**	**38.90**	**48.95**	**43.00**	49.52	**35.80**	**37.30**	**42.26**

**Table 5:** Comparison between the MDAR model, with and without the guidance of the disturbance strategy, in the cross-corpus SER experiments using the IS09 speech feature set. The best result in each task are highlighted in bold.

Rough Emotion Groups	B→ E	E→B	B→C	C→B	E→C	C→E	Average
**Proposed Modification**	**36.52**	**40.29**	**33.10**	**47.32**	31.70	**31.68**	**36.69**
Original Version	36.33	39.89	31.50	46.51	**32.00**	31.50	36.28

## Data Availability

Publicly available datasets were analyzed in this study. This data can be found here: [EmoDB] [http://emodb.bilderbar.info/start.html] (Accessed on 22 May 2022), [eNTERFACE] [http://www.enterface.net/enterface05] (Accessed on 22 May 2022), and [CASIA] [http://www.chineseldc.org] (Accessed on 22 May 2022).
